# A novel magnetic AC/bentonite/Fe_3_O_4_/CeO_2_ nanocomposite catalyst for photocatalytic degradation of persistent chlorinated pesticides in water

**DOI:** 10.1039/d5ra09280a

**Published:** 2026-03-06

**Authors:** Saad S. M. Hassan, Mahmoud Abdelwahab Fathy, Eslam I. El-Aswar, Sabah S. Ibrahim, A. M. Ismael, M. M. Yehia, M. M. Rashad

**Affiliations:** a Department of Chemistry, Faculty of Science, Ain Shams University Abbasia Cairo 11566 Egypt saadsmhassan@yahoo.com Mahmoud.abdelwahab@sci.asu.edu.eg; b Department of Chemistry, School of Engineering Sciences in Chemistry, Biotechnology and Health, KTH Royal Institute of Technology Teknikringen 30 Stockholm SE-100 44 Sweden msaye@kth.se; c National Water Research Center P.O. Box 74 Shoubra El-Kheima 13411 Egypt; d Central Metallurgical Research and Development Institute (CMRDI) P.O. Box 87, Helwan Cairo 11422 Egypt

## Abstract

Persistent organic pesticides (POPs), particularly chlorinated pesticides, represent a critical environmental threat due to their chemical stability, bioaccumulation potential, and resistance to conventional water-treatment methods. Herein, a rapid and cost-effective photocatalytic strategy is developed for the degradation of seventeen structurally diverse POPs in water under ultraviolet (UV) irradiation at ambient temperature. A magnetic heterostructured nanocomposite consisting of activated carbon, derived from sugarcane bagasse, and bentonite, coupled with a magnetite/cerium oxide (Fe_3_O_4_/CeO_2_) nanocomposite, is fabricated *via* a facile ball-milling approach and tested. The material exhibits a strong light absorption, high adsorption capability, high surface activity, and excellent magnetic recoverability. Comprehensive characterization is performed using ultraviolet-visible (UV-vis) spectroscopy, X-ray diffraction (XRD), scanning electron microscopy (SEM), transmission electron microscopy (TEM), and Brunauer–Emmett–Teller (BET) surface analysis. Under optimized conditions (0.1 g L^−1^ catalyst, 20 °C, UV 365 nm, 20 min), removal efficiencies exceeding 90% are achieved for δ-benzene hexachloride (δ-BHC), heptachlor, dichlorodiphenyl trichloroethane (DDT), endrine aldehyde, and methoxychlor, while 73.9–86.6% degradation is recorded for α-BHC, β-BHC, γ-BHC, aldrin, heptachlor epoxide, dichlorodiphenyldichloroethylene (DDE), endrin, dieldrin, dichlorodiphenyldichloroethane (DDD), and endosulfan sulfate. The developed AC/Bentonite/Fe_3_O_4_/CeO_2_ nanocomposite demonstrates some key advantages including high adsorption affinity, fast reaction kinetics, broad pollutant applicability, magnetic separability, and significantly shorter treatment time compared with many of those previously reported using cerium-based photocatalysts. These results highlight the potential of this sustainable catalyst system for efficient remediation of POP-contaminated water.

## Abstract 2

Persistent organic pesticides (POPs), particularly chlorinated pesticides, represent a critical environmental threat due to their chemical stability, bioaccumulation potential, and resistance to conventional water-treatment methods.

## Graphical abstract

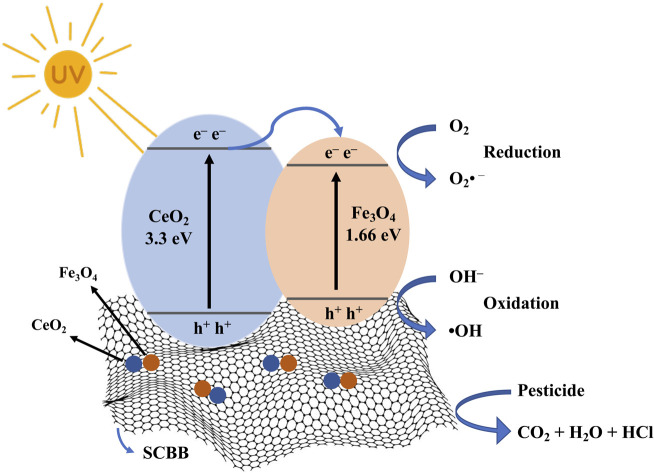

## 1 Introduction

Persistent organic pollutants (POPs), particularly chlorinated pesticides, constitute one of the most hazardous groups of environmental contaminants. Owing to their strong chemical stability, hydrophobicity, and resistance to chemical, biological, and photolytic degradation, these compounds persist and accumulate in various environmental compartments for long periods. Their lipophilic nature facilitates both bioaccumulation in living organisms and biomagnification along the food chain, resulting in severe ecological and public-health concerns. Chronic exposure to chlorinated POPs has been associated with carcinogenicity, endocrine disruption, reproductive dysfunction, immunotoxicity, neurobehavioral disorders, genotoxicity, and developmental abnormalities.^1,2^ Due to their tendency to adsorb onto suspended particulate matter, chlorinated POPs frequently occur and accumulate in aquatic environments. Their widespread presence in surface water, groundwater, and wastewater has prompted strict international regulatory actions. Global frameworks such as the Stockholm Convention and European POP regulations mandate strict control measures including elimination, restriction, and progressive reduction of these pollutants.^3,4^

Several physico-chemical and biological methods have been explored to remove chlorinated pesticides from aquatic systems, including adsorption on activated carbon^5^ and metal–organic frameworks (MOFs),^6^ electrocoagulation,^7^ photocatalytic oxidation,^8^ advanced oxidation technologies (AOTs),^9^ membrane filtration,^10^ ionizing radiation,^11^ and bioremediation using microorganisms.^12^ Although each method offers specific advantages, challenges persist, such as long processing time, high operational cost, secondary waste formation, incomplete mineralization, and limited efficiency toward highly chlorinated and recalcitrant species. Therefore, there remains a critical need for highly efficient, economical, and environmentally benign technologies capable of rapidly degrading many chlorinated POPs under mild operating conditions.

Semiconductor-based photocatalysis has emerged as one of the most promising and sustainable approaches for degrading persistent organic contaminants. Our previous work demonstrated the efficiency of TiO_2_/GO/CuFe_2_O_4_ nanocomposites for the degradation of chlorinated pesticides.^13^ Among semiconductor materials, titanium dioxide (TiO_2_) and cerium dioxide (CeO_2_) were widely employed due to their stability, cost-effectiveness, and low toxicity.^13,14^ CeO_2_ exhibited distinctive physicochemical properties including a tunable band gap (2.6–3.4 eV depending on synthesis method),^15^ remarkable oxygen storage capacity, high redox activity, excellent thermal stability, and facile Ce^4+^/Ce^3+^ transition capability, which enhance photocatalytic oxidation reactions.^16,17^ However, practical limitations such as unfavorable band-edge positions and rapid recombination of charge carriers restrict its standalone photocatalytic efficiency, necessitating structural modification and composite formation.

Previous studies have been investigated with CeO_2_-based photocatalysts for the removal of various pollutants (Table 1), including phenols, dyes, pharmaceuticals, and selected pesticides.^18–27^ Nevertheless, most reported systems suffer from some limitations such as long irradiation periods (30–240 min), high catalyst dosage, narrow target applicability (often one or two analytes), or low mineralization rates. However, the present work offers some significant advantages over many of those reported including the low dose of the catalyst, the short time for the removal process, the removal efficiency, removal selectivity and applicability at ambient temperature.

**Table 1:** Reported performance of different CeO_2_-based photocatalysts for removal of organic contaminants from aqueous media

Photocatalyst	Organic pollutant	Removal time, min	Removal efficiency, %	Ref.
CeO_2_/TiO_2_	Nitrophenol/phenol red	60	97	18
PANI/g-C_3_N_4_/CeO_2_	Diazinon	180	97	19
Fe_3_O_4_/CeO_2_	4-Chlorophenol	60	100	20
CeO_2_–ZnO	Endosulfan	130	55	21
SnO_2_/CeO_2_	Alizarine dye	30	97	22
CeO_2_/Bi_2_WO_6_	Organic dyes	75	54	23
AC/Fe_3_O_4_/CeO_2_	Sulfamethoxazole	120	80	24
Ss/ZnO/CeO_2_	Pesticides	240	75	25
Ag/CeO_2_	Organic dyes	180	96	26
CeO_2_	2,4-Dichloro-phenoxyacetic acid	100	47	27

In parallel to CeO_2_ literature, iron oxide-based photocatalysts—particularly α-Fe_2_O_3_ and Fe_3_O_4_—have been extensively studied due to their natural abundance, low cost, visible-light absorption, and magnetic separability.^28,29^ Several Fe-based photocatalyst systems have demonstrated promising performance for wastewater treatment and for degradation of organic pollutants. α-Fe_2_O_3_ synthesized by sol–gel autocombustion was used for dye degradation under sunlight^30^ and Ag–Fe_2_O_3_ nanohybrids displayed an enhanced visible-light photocatalytic performance.^31^ Moreover, Fe-doped hydroxyapatite encapsulated with alginate has been investigated for wastewater treatment applications.^32^ Fe_3_O_4_-based magnetic composites have attracted attention due to their ability to combine photocatalytic degradation with rapid magnetic recovery and recyclability, which is essential for sustainable operation.^33^ Fe_3_O_4_ has also been reported to exhibit a narrower band gap (1.9–2.2 eV),^34^ which was relevant to its electronic interaction with wide-band-gap semiconductors in composite photocatalysts.

Unlike most reported CeO_2_ or Fe-based photocatalysts that typically dealt with only one or two model contaminants and required extended irradiation periods, the present work introduced a more efficient ternary magnetic nanocomposite. Comprising activated carbon, produced from sugarcane bagasse, bentonite, magnetite (Fe_3_O_4_), and cerium oxide (CeO_2_). In this design, bio-derived activated carbon provided high porosity and strong adsorption affinity, bentonite enhanced pollutant anchoring and structural stability, Fe_3_O_4_ enabled magnetic separation and Fe-mediated redox synergy, while CeO_2_ provided powerful oxidation capability for degrading chlorinated POPs. The SCBB/Fe_3_O_4_/CeO_2_ heterostructured photocatalyst was synthesized *via* a facile ball-milling process and evaluated for the rapid degradation of seventeen different chlorinated POP pesticides in water under UV irradiation at ambient temperature. The system demonstrated fast kinetics (≤20 min), high removal efficiency, operational sustainability, magnetic recovery, cost-effectiveness and environmentally sustainable approach for chlorinated POP remediation.

## 2 Experimental

### 2.1. Equipment

A NOVA touch® LX2 surface area and pore-size analyzer (Quantachrome Instruments, USA) was used to determine the surface area and pore characteristics of the nanocomposite using the Brunauer–Emmett–Teller (BET) method with N_2_ adsorption–desorption isotherms at 77 K. UV-vis-NIR diffuse-reflectance spectra were recorded using a Jasco V-570 spectrophotometer (Jasco, Japan). The surface morphology and microstructure were examined using field-emission scanning electron microscopy (FE-SEM, FEI, USA) and transmission electron microscopy (TEM, JEOL 2010F, Japan). Crystal structures were characterized using a Philips PW/103 X-ray diffractometer with Cu-Kα radiation (*λ* = 1.5406 Å) operated at 40 kV and 40 mA over a 2*θ* range of 0–70°. Room-temperature magnetic hysteresis measurements were obtained using a vibrating sample magnetometer (VSM, MDK, Germany) over an applied magnetic field of ±10 kOe. Photocatalytic experiments were performed under UV illumination using a 150 W xenon arc lamp (CHF-XQ-500W, Beijing Changtuo Co., Ltd, China) at 365 nm.

Chemical analysis of pesticide residues was carried out using an Agilent 7890A GC system coupled to a triple-quadrupole mass spectrometer (5975C) and controlled by MassHunter software. A 7693 autosampler and an HP-5MS Ultra Inert capillary column (30 m × 250 µm × 0.25 µm) were employed. Helium served as the carrier gas at 1.0 mL min^−1^. The oven program was set as follows: 80 °C for 1 min, ramp to 175 °C at 30 °C min^−1^ and hold for 4 min, then increased to 225 °C at 3 °C min^−1^ with a 6-min hold, giving a 20-min effective runtime and a 30-min total analysis time.

### 2.2. Materials and reagents

Sugarcane bagasse (SCB) was collected from a local market in Cairo, Egypt. Bentonite was obtained from CMB Company (Wadi El-Natroun Industrial Zone, Egypt). Hydrochloric acid (HCl, 33%, ADWIC, Egypt) and sodium hydroxide (NaOH) were used for pH adjustment. A certified organochlorine pesticide (OCP) standard mixture was purchased from AccuStandard Inc. (New Haven, USA). Dichloromethane (98%) and *n*-hexane (98%) of HPLC grade (LiChrosolv) were supplied by Merck (Darmstadt, Germany). Magnetite (Fe_3_O_4_, < 20 µm particle size), cerous nitrate [Ce(NO_3_)_3_·6H_2_O, 99%], hydrogen peroxide (H_2_O_2_, 30% w/w), phosphoric acid (H_3_PO_4_, 40% w/v), and glycine (≥99%, HPLC grade) were obtained from Sigma-Aldrich.

### 2.3. Preparation of sugarcane-bagasse/bentonite composite (SCBB)

The sugarcane bagasse was thoroughly washed with distilled water to remove surface impurities, then dried at 105 °C overnight to eliminate moisture. The dried biomass was ground and mixed with bentonite and polyurethane in a 2 : 1 : 1 weight ratio, respectively to obtain (SCBBPU precursor). The mixture was homogenized and heated at 200 °C for 30 min.

A 50 g portion of the dried SCBBPU was soaked in 200 mL of 40% (w/v) H_3_PO_4_ for 24 h under anaerobic conditions. The mixture was then heated to 85 °C with stirring for 1 h and subsequently carbonized in a closed system. The temperature was increased to 500 °C at a ramp rate of 4 °C min^−1^ and held for 3 h. After cooling, the product was washed with distilled water to remove excess acid, dried at 120 °C for 3 h, and finally activated at 650 °C in a muffle furnace. The resulting SCBB composite was stored in airtight glass containers until use.

### 2.4. Synthesis of cerium oxide (CeO_2_) nanoparticles

CeO_2_ nanoparticles were synthesized *via* a sol–gel auto-combustion route.^35^ Cerous nitrate was dissolved in distilled water and mixed with a stoichiometric amount of glycine, followed by the addition of 10% (w/v) hydrogen peroxide as an oxidizing agent. The solution was gently heated with continuous stirring until a viscous gel was formed. The resulting gel was dried at 100 °C overnight, then calcined in a muffle furnace at 400 °C for 2 h to obtain crystalline CeO_2_ nanoparticles.

### 2.5. Preparation of SCBB/Fe_3_O_4_/CeO_2_ nanocomposite

The nanocomposite catalyst was prepared by combining SCBB (1.5 g), magnetite (0.5 g), and CeO_2_ nanoparticles (3.5 g) with zirconia grinding media (20 g) in a milling chamber. The ball-to-powder weight ratio was maintained at the ratio 10 : 1. The mixture was subjected to high-energy ball milling at 550 rpm for 3 h to obtain a homogeneous SCBB/Fe_3_O_4_/CeO_2_ nanocomposite.

### 2.6. Photocatalytic degradation of chlorinated pesticides

The photocatalytic performance of the prepared nanocomposite was evaluated using a standard organochlorine pesticide mixture containing 17 compounds. In a typical experiment, 0.1 g of the catalyst was dispersed in 50 mL of pesticide test solution, to which 1.0 mL of the standard mixture was added. The pH was adjusted to 3.0 ± 0.1 and the suspension was stirred in the dark for 10 min to ensure uniform dispersion and to allow adsorption–desorption pre-equilibrium between the pesticides and the catalyst surface, in line with commonly adopted photocatalytic degradation protocols.^36,37^ The reaction mixture was then irradiated under UV light using a xenon arc lamp (365 nm) for 20 min. Photocatalytic experiments were conducted under ambient air without oxygen injection, and continuous magnetic stirring was maintained during irradiation at approximately 600 rpm to ensure homogeneous suspension. The 150 W xenon lamp was positioned vertically above the reactor at a fixed distance of approximately 10 cm, with an irradiance of approximately 50 mW cm^−2^ at 365 nm. A blank experiment was carried out under identical conditions without the standard pesticide mixture. A photolysis control test (pesticide solution + UV only) was additionally performed under the same irradiation conditions in the absence of the SCBB/Fe_3_O_4_/CeO_2_ nanocomposite to assess the contribution of direct photolysis. In all photocatalytic experiments, the initial pesticide concentrations in the reaction medium were dictated by the certified standard mixture, resulting in analyte levels in the range of 0.5–2.0 mg L^−1^. Reaction aliquots were collected at *t* = 20 min and analyzed by GC-MS/MS for quantification.

Following irradiation, residual pesticides were extracted by liquid–liquid extraction according to method 6630B, and quantified by GC-MS/MS as previously described.^13^ The degradation efficiency was calculated using eqn (1):^38^1
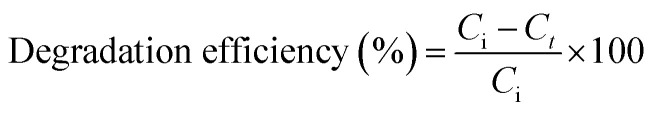
where *C*_i_ and *C*_*t*_ are the initial (control sample concentration) and final (remaining) pesticide concentrations (mg L^−1^), respectively.

## 3 Results and discussion

A novel nanocomposite photocatalyst consisting of activated carbon, derived from sugarcane bagasse (SCB), bentonite (B), Fe_3_O_4_, and CeO_2_ was successfully synthesized, characterized, and evaluated for the degradation of chlorinated pesticides.

### 3.1. Characterization of SCBB/Fe_3_O_4_/CeO_2_ nanocomposite

#### 3.1.1. Transmission electron microscopy (TEM)

**Fig. 1:**
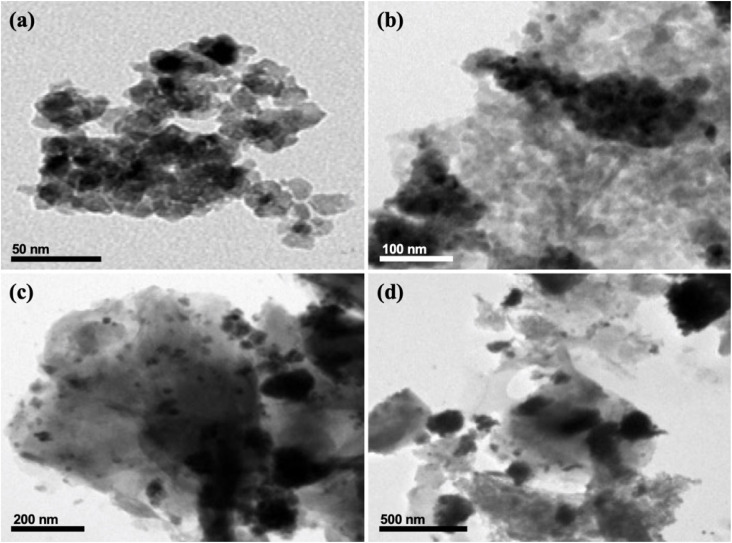
TEM images of (a) CeO_2_ nanoparticles^39^ and (b–d) SCBB/Fe_3_O_4_/CeO_2_ nanocomposite at different magnifications, showing nanoscale particle morphology and uniform distribution of metal oxides within the carbon–bentonite matrix.

TEM analysis was performed to investigate the morphology and particle distribution of CeO_2_ nanoparticles and the SCBB/Fe_3_O_4_/CeO_2_ nanocomposite. As shown in Fig. 1a, CeO_2_ nanoparticles exhibited a nearly spherical morphology with nanoscale dimensions. The SCBB/Fe_3_O_4_/CeO_2_ nanocomposite (Fig. 1b–d) revealed agglomerated nanosized particles uniformly anchored onto the carbon–bentonite matrix, confirming successful incorporation and dispersion of CeO_2_ and Fe_3_O_4_ into the nanocomposite structure.

#### 3.1.2. Scanning electron microscopy (SEM)

**Fig. 2:**
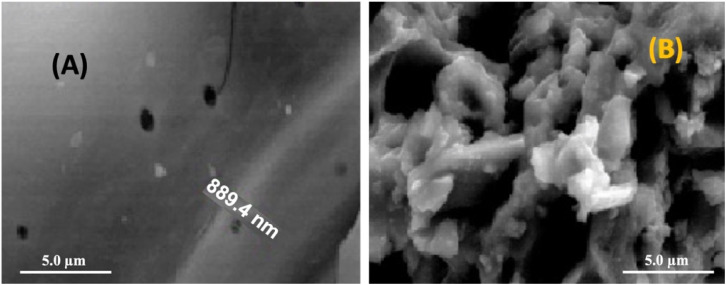
SEM micrographs of (A) raw SCB and (B) SCBB composite illustrating the development of a porous surface structure following chemical activation.

SEM analysis was used to examine the surface morphology of raw sugarcane bagasse (SCB) and the modified SCBB composite. As shown in Fig. 2a, the raw SCB exhibited compact and stacked cellulose layers with limited visible porosity. Following chemical activation and incorporation of bentonite (Fig. 2b), the SCBB structure became highly porous and rough, indicating successful surface modification and pore development.

The formation of this porous network was attributed to phosphoric acid activation and bentonite incorporation, which enhance surface area and improve the material's capacity for pesticide adsorption—an essential step prior to photocatalytic degradation.

#### 3.1.3. X-ray diffraction (XRD) analysis

**Fig. 3:**
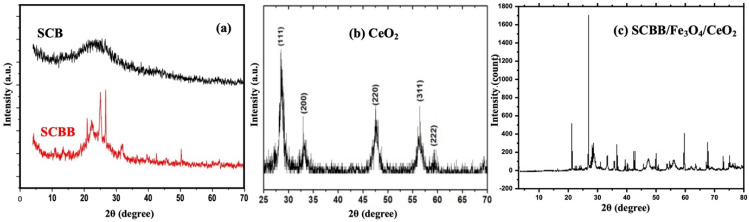
XRD patterns of (a) raw sugarcane bagasse (SCB) and activated SCBB composite, (b) CeO_2_ nanoparticles, and (c) SCBB/Fe_3_O_4_/CeO_2_ nanocomposite.

XRD analysis was performed to determine the crystalline phases and structural features of the raw materials and the synthesized SCBB/Fe_3_O_4_/CeO_2_ nanocomposite. As shown in Fig. 3a, raw sugarcane bagasse (SCB) exhibited a broad diffraction hump, indicating its predominantly amorphous nature due to the disordered cellulose–lignin matrix. After chemical activation and modification, the SCBB pattern displayed sharper reflections associated with the formation of graphitic carbon domains, along with a peak near ∼22.4°, corresponding to silica originating from bentonite minerals.^40^

The crystalline phase of CeO_2_ nanoparticles was confirmed by the presence of distinct diffraction peaks at 2*θ* ≈ 28.8°, 32.9°, 47.6°, and 56.6°, which correspond to (111), (200), (220), and (311) planes of the fluorite cubic CeO_2_ structure, as shown in Fig. 3b and consistent with JCPDS Card No. 34-0394.^41^ In the SCBB/Fe_3_O_4_/CeO_2_ nanocomposite (Fig. 3c), characteristic peaks of Fe_3_O_4_ at 2*θ* ≈ 30.2°, 35.5°, 43.3°, 57.2°, and 62.8°, along with CeO_2_ reflections, were developed and clearly observed, confirming their successful incorporation. The Fe_3_O_4_ reflections appeared at (220), (311), (400), (511), and (440) planes, were in a good agreement with JCPDS Card No. 19-0629,^42^ confirming the presence of the magnetite phase in the prepared composite. Additional reflections attributed to carbon and aluminosilicate phases of bentonite further supported the formation of a multi-component heterostructure.

These results verified the coexistence of CeO_2_, Fe_3_O_4_, carbon, and bentonite phases within the composite, demonstrating successful synthesis without compromising the crystalline integrity of the incorporated metal oxides. The clear peak definition also indicated nanoscale crystallite dimensions and maintained structural stability, supporting the photocatalytic activity and durability of the composite.

#### 3.1.4. Ultraviolet-visible (UV-vis) absorption spectroscopy

**Fig. 4:**
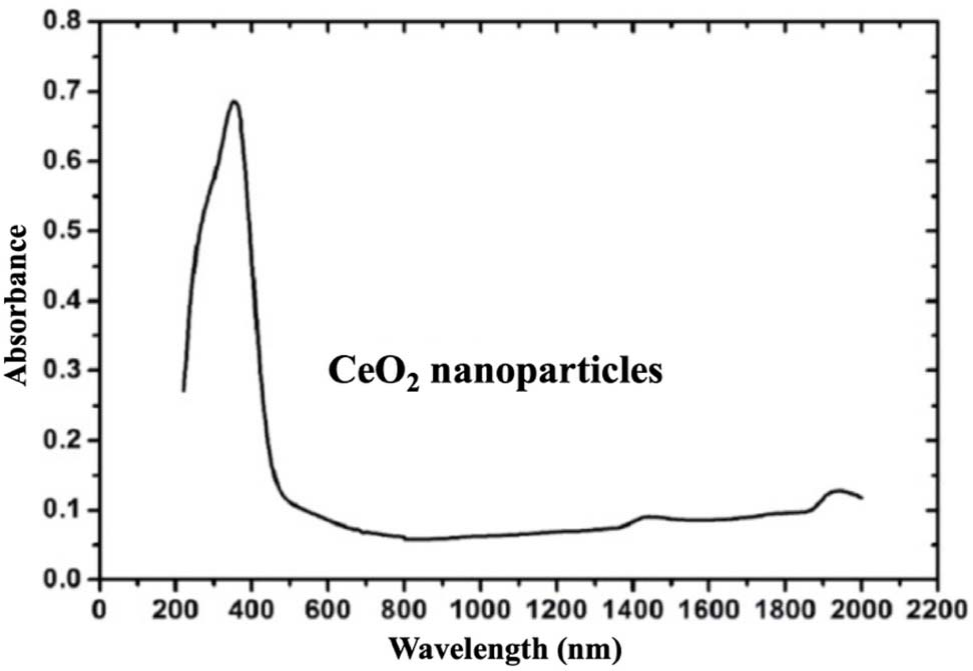
UV-vis-NIR absorption spectrum of CeO_2_ nanoparticles recorded in the wavelength range of 200–2000 nm.

UV-vis absorption spectroscopy was conducted to investigate the optical properties of the synthesized CeO_2_ nanoparticles. As illustrated in Fig. 4, the CeO_2_ sample exhibited a strong and broad absorption band in the UV region, with an intense peak appeared near ∼400 nm. This characteristic absorption was attributed to charge-transfer transitions from O^2−^ to Ce^4+^ ions, a typical for CeO_2_ nanomaterials and agreed with the previously reported studies.^43^

In addition, a slight absorption tail extending toward the visible region and associated with defect-related sub-bandgap states, was commonly linked to oxygen vacancies and Ce^3+^ centres in ceria-based systems.^44^ Such defects are known to play a key role in enhancing photocatalytic performance by facilitating charge separation and broadening the light-absorption range.^39^ These optical features further supported the applicability of CeO_2_ in photocatalytic systems operating under UV illumination, and indicating the possible contribution of defect states to light absorption.

#### 3.1.5. Surface area and pore structure analysis

**Fig. 5:**
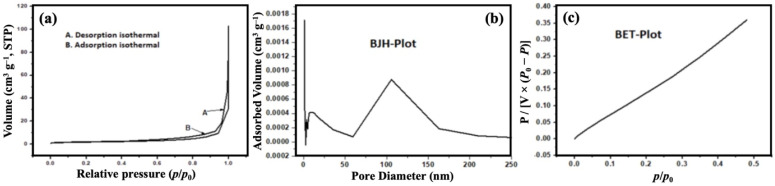
N_2_ adsorption–desorption analysis of SCBB/Fe_3_O_4_/CeO_2_ nanocomposite: (a) adsorption–desorption isotherm, (b) BJH pore-size distribution, and (c) BET linear plot.

Nitrogen adsorption–desorption analysis was performed to explore the textural properties of the SCBB/Fe_3_O_4_/CeO_2_ nanocomposite. As shown in Fig. 5a–c, the material exhibited a type-IV isotherm with a clear H_3_-type hysteresis loop, characteristic of mesoporous solids according to IUPAC classification.^45^ BET analysis revealed a specific surface area of 6.54 m^2^ g^−1^ and a total pore volume of 0.042 cm^3^ g^−1^, as listed in Table 2, indicating the presence of accessible pore channels formed during phosphoric-acid activation and bentonite incorporation. Although the measured specific surface area appeared relatively low, similar values have been reported for hybrid bentonite systems, where clay platelets partially shield nitrogen-accessible micro- and mesopores, resulting in reduced external surface area.^46,47^ Metal oxide loading is also known to reduce the apparent surface area of carbonaceous supports; for instance, Fe_3_O_4_–CeO_2_/activated carbon composites showed a substantial reduction in BET surface area compared to pristine activated carbon due to oxide nanoparticle deposition and pore blocking.^48^ Furthermore, Fe(iii)-modified bentonites have demonstrated reduced nitrogen accessibility and densified interlayer structures, supporting the observed decrease in surface area in mineral–oxide system. These observations indicated that the relatively low BET surface area of the SCBB/Fe_3_O_4_/CeO_2_ composite pointed to a hybrid architecture of bentonite + biochar + metal oxides, rather than to a deficiency in the porosity or reactivity.

**Table 2:** BET surface area and BJH pore-structure parameters of SCBB/Fe_3_O_4_/CeO_2_ nanocomposite obtained from N_2_ adsorption–desorption analysis (77.36 K; sample mass = 0.5447 g; manifold volume = 33.951 cm^3^; free space = 33.848 cm^3^)

Parameter	Value ± SD
**BETanalysis**	
*V*_m_ (cm^3^ STP g^−1^)	1.5034 ± 0.0752
BET surface area, *a*_s_ (m^2^ g^−1^)	6.5435 ± 0.3272
*C* constant	86.539 ± 4.327
Total pore volume, *V*_t_ (cm^3^ g^−1^)	0.04229 ± 0.00211
Average pore diameter (nm)	25.853 ± 1.293

**BJH analysis**	
Pore volume, *V*_p_ (cm^3^ g^−1^)	0.09815 ± 0.00491
Peak pore diameter (nm)	1.0454 ± 0.0523
Pore surface area (m^2^ g^−1^)	8.5861 ± 0.4293
Average pore diameter (nm)	45.726 ± 2.286
Median pore diameter (nm)	85.672 ± 4.284

The BJH pore-size distribution (Fig. 5b) further confirmed the predominantly mesoporous nature of the composite, with an average pore diameter of ≈45.7 nm. Such mesoporous architecture enhanced the adsorption and diffusion of pesticide molecules toward active sites, thereby supporting efficient photocatalytic degradation. The combined effect of carbonaceous porosity and bentonite templating provided a favourable textural framework for improved pollutant uptake and photocatalytic activity.

#### 3.1.6. Magnetic properties (VSM analysis)

**Fig. 6:**
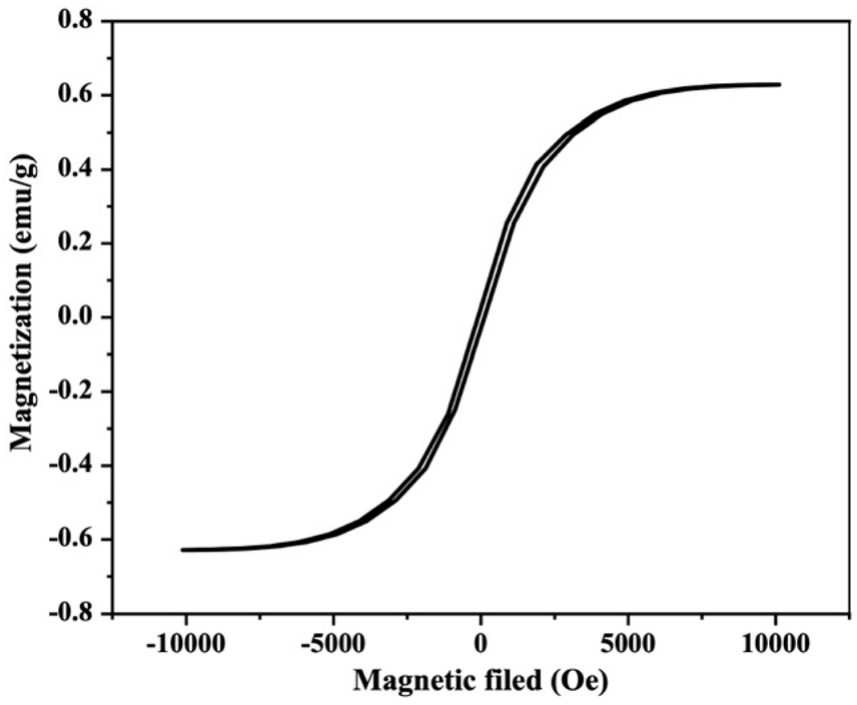
VSM hysteresis loop of the SCBB/Fe_3_O_4_/CeO_2_ nanocomposite measured at room temperature within an applied magnetic field of ±10 kOe.

The magnetic properties of the SCBB/Fe_3_O_4_/CeO_2_ nanocomposite were evaluated using vibrating sample magnetometry (VSM) at room temperature over an applied magnetic field of ±10 kOe. As shown in Fig. 6, the M–H hysteresis loop exhibited soft magnetic behaviour characterized by low coercivity and small remanent magnetization. The saturation magnetization (*M*_s_) reached approximately 0.63 emu g^−1^, which was considerably lower than that of bulk magnetite due to dilution of the magnetic Fe_3_O_4_ phase by non-magnetic SCBB and CeO_2_ components.

Despite the modest *M*_s_, the nanocomposite responded rapidly to an external magnet, allowing complete separation from aqueous suspension within ∼15–20 s. This rapid magnetic recovery eliminated the need for centrifugation or filtration during catalyst harvesting and facilitated solid-phase recycling, offering a practical advantage for sustainable water-treatment applications.

### 3.2. Photodegradation of chlorinated pesticides using SCBB/Fe_3_O_4_/CeO_2_ nanocomposite

The efficiency of the synthesized SCBB/Fe_3_O_4_/CeO_2_ nanocomposite toward the removal and photocatalytic degradation of seventeen chlorinated pesticides (0.5–2.0 mg L^−1^) was evaluated under dark and UV-irradiation conditions. A recent review reported that the concentrations of organochlorine pesticides in rivers worldwide during 2014–2024 ranged from 0.4 to 37 × 10^5^ ng L^−1^ (*i.e.*, up to 3.7 mg L^−1^), depending on location, pollution sources, and seasonal variation.^49^ In addition, concentrations as low as 0.4 µg L^−1^ have been detected in certain local wastewater samples.^50^ Therefore, the selected concentration range (0.5–2.0 mg L^−1^) falls within the upper levels reported for highly contaminated river systems and allows reliable analytical quantification and performance evaluation.

Preliminary adsorption–equilibrium tests indicated that extending the dark stirring period from 10 to up to 30 min did not lead to noticeable changes in removal efficiencies, suggesting that the adsorption–desorption equilibrium was reached rapidly under the applied conditions. As presented in Table 3, the catalyst exhibited a noticeable removal capability in the absence of light, where adsorptive removal efficiencies ranged from 13.5 ± 0.6% to 75.2 ± 3.9% after 20 min. This behaviour highlighted the strong affinity of the sugarcane-bagasse-derived activated carbon, in combination with bentonite, toward chlorinated pesticides due to the presence of accessible adsorption sites and surface functional groups,^51^ which collectively facilitated pre-concentration of the pollutants at the catalyst surface prior to photocatalytic activation.

**Table 3:** Retention times (*R*_t_) and removal/degradation efficiencies (mean ± SD) of chlorinated pesticides following treatment with SCBB/Fe_3_O_4_/CeO_2_ nanocomposite under dark and UV irradiation. Initial pesticide concentrations in the reaction medium were in the range of 0.5–2.0 mg L^−1^, and all removal values correspond to end-point measurements at *t* = 20 min

Pesticide	Retention time (*R*_t_), min	Removal % (dark)	Photocatalytic degradation, % (UV)
α-BHC	8.96	40.3 ± 2.1	78.8 ± 3.4
β-BHC	9.87	39.1 ± 1.9	79.9 ± 2.8
γ-BHC	10.11	36.2 ± 1.7	83.4 ± 3.1
δ-BHC	11.02	40.8 ± 2.3	74.2 ± 2.6
Heptachlor	13.03	37.9 ± 1.5	86.1 ± 3.7
Aldrin	14.60	60.9 ± 3.4	84.6 ± 2.5
Heptachlor epoxide	16.58	29.2 ± 1.2	57.3 ± 1.8
Endosulfan I	18.42	75.2 ± 3.9	97.3 ± 2.9
Dieldrin	19.79	51.9 ± 2.4	93.0 ± 3.6
DDE	19.97	35.7 ± 1.6	77.9 ± 2.3
Endrin	20.92	68.5 ± 3.1	92.3 ± 2.7
Endosulfan II	21.46	66.5 ± 2.8	60.0 ± 1.9
DDD	22.17	56.0 ± 2.0	86.6 ± 3.2
Endrin aldehyde	22.52	25.2 ± 1.1	78.9 ± 2.4
Endosulfan sulfate	23.83	13.5 ± 0.6	90.5 ± 3.8
DDT	24.21	36.2 ± 1.4	73.9 ± 2.6
Methoxychlor	28.05	26.6 ± 1.3	93.7 ± 4.1

It should be noted that the degradation values presented in Table 3 represent end-point measurements at *t* = 20 min, which was selected based on preliminary GC-MS/MS monitoring. Extending the irradiation beyond this interval did not substantially change the residual pesticide profiles under the applied conditions.

Upon exposure to UV irradiation (365 nm), the composite catalyst demonstrated a significant enhancement in performance, achieving photocatalytic degradation efficiencies ranging from 57.3 ± 1.8% (for heptachlor epoxide) to 97.7 ± 4.1% (for methoxychlor) under the same conditions. Many of the tested chlorinated pesticides were decomposed with efficiencies > 90% (endosulfan-I 97.3 ± 2.9%, dieldrin, 93.0 ± 3.6%, endrin, 92.3 ± 2.7%, endosulfan sulfate 90.5 ± 3.8%). This pronounced increase in the decomposition under UV illumination confirmed that the SCBB/Fe_3_O_4_/CeO_2_ composite operated *via* a dual-stage mechanism, where the initial adsorption enriched pesticide molecules on the catalyst surface, followed by rapid photodegradation through electron–hole-driven oxidative pathways.

The synergistic contribution of the nanocomposite components was central to this enhanced performance. The activated carbon matrix provided extensive adsorption sites,^52,53^ whereas bentonite enhanced structural stability and surface interaction.^51,54^ Fe_3_O_4_ enabled magnetic recovery and participated in redox mediation, which not only facilitated catalyst separation but also allowed the material to be reused while maintaining its photocatalytic performance for up to four consecutive cycles. On the other hand, CeO_2_ facilitated efficient charge separation and promoted reactive oxygen species (ROS) generation through Ce^4+^/Ce^3+^ cycling. Such synergy accelerated radical-mediated oxidation and minimized charge recombination. This behaviour aligned with a previously reported mechanisms where adsorption-driven enrichment enhanced photocatalytic decomposition efficiency in hybrid carbon-ceria materials.^55^

**Fig. 7:**
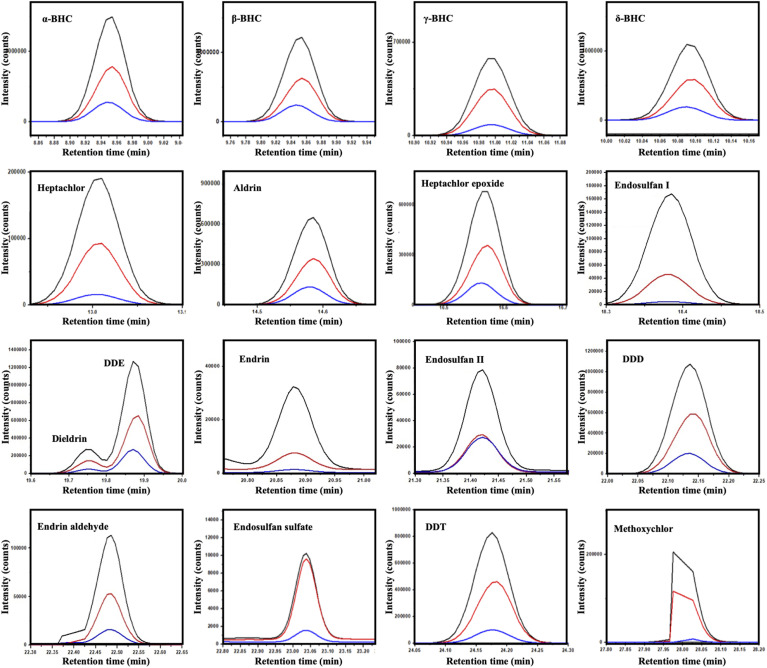
GC-MS chromatograms of seventeen chlorinated pesticides: black trace (standard), red trace (dark treatment), and blue trace (UV treatment) using the SCBB/Fe_3_O_4_/CeO_2_ nanocomposite at ambient temperature.

GC-MS chromatograms (Fig. 7) further validated the photocatalytic degradation process, showing a substantial reduction or disappearance of characteristic pesticide peaks following treatment, while exposure to the UV-only (photolysis control) did not lead to measurable attenuation of pesticide peaks, confirming that direct photolysis was negligible under the applied irradiation conditions. This negligible photolysis was consistent with the classification of these chlorinated pesticides as persistent organic pollutants (POPs), which are known for their chemical and photochemical stability due to extensive chlorination and limited absorption in the UVA range.

### 3.3. Comparison of photocatalytic degradation performance with other nanocomposites

Although various photocatalysts have been previously investigated for pesticide removal, comprehensive degradation studies covering a wide range of structurally different chlorinated pesticides remained scarce; most reported works frequently focus on one or two compounds only. Accordingly, a comparative photocatalytic evaluation was conducted for the degradation of seventeen common chlorinated pesticides using three nanocomposite catalysts synthesized and used in our laboratory under identical UV-irradiation conditions for 20 min. The performance of the present SCBB/Fe_3_O_4_/CeO_2_ nanocomposite was benchmarked against TiO_2_/GO/CuFe_2_O_4_ (ref. 13) and Mo-TiO_2_/GO/Fe_3_O_4_.^56^ The results, presented in Table 4, revealed that each catalyst exhibited a preferential degradation pattern toward specific pesticide classes.

**Table 4:** Photocatalytic degradation of some chlorinated pesticides after 20 min UV exposure using three nanocomposite catalysts

Pesticide	Retention time (*R*_t_), min	Photocatalytic degradation % (UV)		
		TiO_2_/GO/CuFe_2_O_4_ (ref. 13)	Mo-TiO_2_/GO/Fe_3_O_4_ (ref. 56)	SCBB/Fe_3_O_4_/CeO_2_ [present work]
α-BHC	8.96	92.2	83.8	78.8 ± 3.4
β-BHC	9.87	93.4	80.9	79.9 ± 2.8
γ-BHC	10.11	85.4	82.4	83.4 ± 3.1
δ-BHC	11.02	70.6	90.7	74.2 ± 2.6
Heptachlor	13.03	71.7	90.7	86.1 ± 3.7
Aldrin	14.60	95.9	81.5	84.6 ± 2.5
Heptachlor epoxide	16.58	89.2	82.8	57.3 ± 1.8
Endosulfan I	18.42	86.5	93.5	97.3 ± 2.9
Dieldrin	19.79	84.8	69.9	93.0 ± 3.6
DDE	19.97	96.5	79.9	77.9 ± 2.3
Endrin	20.92	89.2	94.3	92.3 ± 2.7
Endosulfan II	21.46	79.1	55.6	60.0 ± 1.9
DDD	22.17	76.1	83.6	86.6 ± 3.2
Endrin aldehyde	22.52	70.4	58.4	78.9 ± 2.4
Endosulfan sulfate	23.83	32.4	88.1	90.5 ± 3.8
DDT	24.21	59.6	89.1	73.9 ± 2.6
Methoxychlor	28.05	42.7	93.6	93.7 ± 4.1

It can be seen that TiO_2_/GO/CuFe_2_O_4_ nanocomposite demonstrated superior activity toward α-BHC, β-BHC, γ-BHC, aldrin, heptachlor epoxide, endosulfan II, and DDE, giving degradation efficiencies in the range of 79.1–96.5%. In contrast, Mo-TiO_2_/GO/Fe_3_O_4_ system showed enhanced degradation of δ-BHC, heptachlor, endrin, and DDT, with efficiencies ranging between 89.1–94.3%. The present suggested nanocomposite catalyst (SCBB/Fe_3_O_4_/CeO_2_) exhibited the highest efficiency (78.9–97.3%) toward endosulfan I, dieldrin, DDD, endrin aldehyde, endosulfan sulfate, and methoxychlor.

The preferential degradation patterns observed in Table 4 can be rationalized by considering structure-performance correlations. For instance, highly hydrophobic and heavily chlorinated pesticides such as endosulfan I, dieldrin and DDD tend to exhibit stronger affinity toward carbonaceous and clay-containing systems due to non-polar and van der Waals interactions, promoting pre-adsorption and facilitating subsequent photocatalytic conversion. In contrast, TiO_2_-based photocatalysts, which possess more hydrophilic surfaces and different band edge positions, have been reported to preferentially degrade less hydrophobic or sterically smaller organochlorines. Therefore, both the physicochemical properties of the pesticides (*e.g.* hydrophobicity, molecular size and degree of chlorination) and the surface chemistry and band structure of the catalysts collectively contributed to the observed differences in degradation selectivity among the three systems.

### 3.4. Mechanism of action of SCBB/Fe_3_O_4_/CeO_2_ nanocomposite

**Scheme 1:**
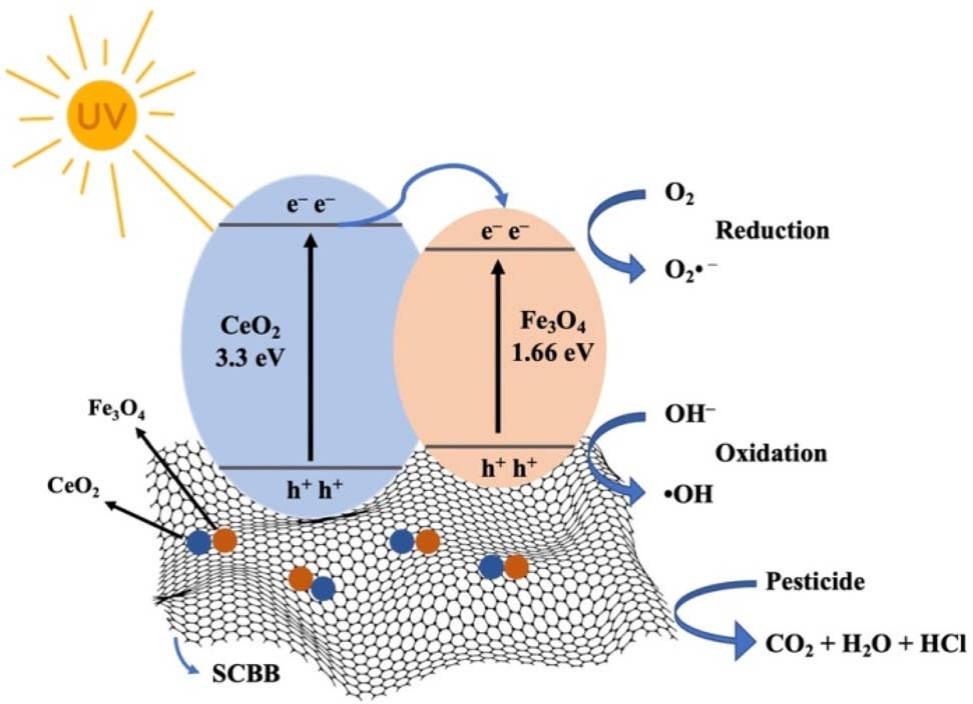
Schematic mechanism for photocatalytic degradation of chlorinated pesticides using SCBB/Fe_3_O_4_/CeO_2_ nanocomposite under UV irradiation.

The photocatalytic activity of the SCBB/Fe_3_O_4_/CeO_2_ nanocomposite toward chlorinated pesticides under UV irradiation proceeded through a combined photocatalytic and Fenton-like pathway, as illustrated in Scheme 1. Upon UV exposure, electrons were promoted from the valence band (VB) to the conduction band (CB), leaving their corresponding holes in the VB. The generated charge carriers migrated efficiently between Fe_3_O_4_ and CeO_2_ due to their favourable band potentials, thereby suppressing electron–hole recombination. The holes reacted with water molecules or surface hydroxyl groups to generate hydroxyl radicals (˙OH),^57^ while the electrons reduced dissolved oxygen to superoxide radicals (O_2_˙^−^). Both species initiated oxidation and cleavage of chlorinated pesticide molecules. O_2_˙^−^ can further react with protons to generate additional ˙OH, intensifying the oxidative environment.

Simultaneously, Fe^2+^/Fe^3+^ species in the Fe_3_O_4_ phase and Ce^4+^/Ce^3+^ cycling in CeO_2_ (E° Ce^4+^/Ce^3+^ = 1.44 V *vs.* E° Fe^2+^/Fe^3+^ = 0.77 V) promoted Fenton-like reactions in which Fe^2+^ reacted with H_2_O_2_ to produce surface-bound hydroxyl radicals (˙OH_ads).^58^ CeO_2_ facilitated regeneration of Fe^2+^ from Fe^3+^, sustaining the catalytic cycle. A fraction of Fe ions may also diffuse into solution, contributing to homogeneous Fenton reactions that generate free hydroxyl radicals (˙OH_free). These reactive oxygen species mineralized pesticide molecules into CO_2_, H_2_O, and HCl.

## 4 Conclusion

In this study, a cost-effective and sustainable magnetic AC/Bentonite/Fe_3_O_4_/CeO_2_ nanocomposite was successfully synthesized from sugarcane bagasse and evaluated as a multifunctional adsorbent-photocatalyst for the removal of seventeen persistent chlorinated pesticides from water. Comprehensive characterization confirmed the formation of a mesoporous, heterostructured material with enhanced surface properties and efficient light-absorption capability. Under mild conditions (0.1 g L^−1^ catalyst, UV 365 nm, ambient temperature), the nanocomposite demonstrated rapid degradation performance, achieving up to ∼97% removal within only 20 min and showing significant adsorption activity in dark conditions. The superior efficiency can be attributed to the synergistic contribution of activated carbon, bentonite, Fe_3_O_4_, and CeO_2_, enabling strong pollutant adsorption, efficient charge separation, and enhanced radical generation *via* Ce^4+^/Ce^3+^ and Fe^3+^/Fe^2+^ redox cycling. Compared to previously reported systems, the proposed catalyst offered faster kinetics, broader applicability, and magnetic recoverability. These characteristics demonstrated the strong potential of the suggested catalyst for the treatment of POPs-contaminated water.

## Ethical approval

Ethical approval was obtained from the Ethical Committee (EC) of the Faculty of Science, Ain Shams University, Cairo, Egypt, for the experimental procedures conducted in this study.

## Author contributions

Saad S.M. Hassan: supervision, conceptualization, validation, writing reviewing & editing. Mahmoud Abdelwahab Fathy: conceptualization, resources, methodology, investigation, formal analysis, data curation, visualization, validation, writing – original draft, writing – reviewing & editing. Eslam I. El-Aswar: resources, investigation. Sabah S. Ibrahim: resources, investigation. A. M. Ismael: investigation, formal analysis, visualization. M. M. Yehia: resources, investigation. M. M. Rashad: supervision, conceptualization, validation.

## Conflicts of interest

The authors declare no competing interests.

## Data Availability

Data will be made available on request.

## References

[cit1] Alharbi O. M., Khattab R. A., Ali I. (2018). Health and environmental effects of persistent organic pollutants. J. Mol. Liq..

[cit2] Kumari K. , Balbudhe S. and Singh A., Effects of Persistent Organic Pollutants (POPs) on Human Health, Persistent Organic Pollutants, CRC Press, 2021, pp. 91–122

[cit3] Fiedler H., Kallenborn R., Boer J. D., Sydnes L. K. (2019). The Stockholm convention: a tool for the global regulation of persistent organic pollutants. Chem. Int..

[cit4] Regulation E. , 1021 of the European Parliament and of the Council of 20 June 2019 on Persistent Organic Pollutants, European Union, 2019

[cit5] Ighalo J. O., Yap P.-S., Iwuozor K. O., Aniagor C. O., Liu T., Dulta K., Iwuchukwu F. U., Rangabhashiyam S. (2022). Adsorption of persistent organic pollutants (POPs) from the aqueous environment by nano-adsorbents: A review. Environ. Res..

[cit6] Naghdi S., Shahrestani M. M., Zendehbad M., Djahaniani H., Kazemian H., Eder D. (2023). Recent advances in application of metal-organic frameworks (MOFs) as adsorbent and catalyst in removal of persistent organic pollutants (POPs). J. Hazard. Mater..

[cit7] Titchou F. E., Zazou H., Afanga H., El Gaayda J., Ait Akbour R., Hamdani M. (2021). Removal of persistent organic pollutants (POPs) from water and wastewater by adsorption and electrocoagulation process. Groundw. Sustain. Dev..

[cit8] Pascariu P., Cojocaru C., Ciornea V., Romanitan C., Serban A. B. (2024). Visible light-responsive Ce-doped ZnO ceramic nanostructures as effective photocatalysts for removal of persistent organic pollutants from contaminated waters. Mater. Today Sustain..

[cit9] Clímaco Cunha I. L., Machado P. G., de Oliveira Ribeiro C., Kulay L. (2024). Bibliometric analysis of Advanced Oxidation Processes studies with a focus on Life Cycle Assessment and Costs. Environ. Sci. Pollut. Res..

[cit10] Mhlongo S. A., Sibali L. L., Ndibewu P. P. (2023). Some aspects of the synthesis, characterization and modification of poly (ether) sulfone polymeric membrane for removal of persistent organic pollutants in wastewater samples. J. Water Chem. Technol..

[cit11] Trojanowicz M. (2020). Removal of persistent organic pollutants (POPs) from waters and wastewaters by the use of ionizing radiation. Sci. Total Environ..

[cit12] Mateescu C., Lungulescu E.-M., Nicula N.-O. (2024). Effectiveness of Biological Approaches for Removing Persistent Organic Pollutants from Wastewater: A Mini-Review. Microorganisms.

[cit13] Ismael A., El-Shazly A., Gaber S., Rashad M., Kamel A., Hassan S. S. M. (2020). Novel TiO_2_/GO/CuFe_2_O_4_ nanocomposite: a magnetic, reusable and visible-light-driven photocatalyst for efficient photocatalytic removal of chlorinated pesticides from wastewater. RSC Adv..

[cit14] Montini T., Melchionna M., Monai M., Fornasiero P. (2016). Fundamentals and catalytic applications of CeO_2_-based materials. Chem. Rev..

[cit15] Sharma D., Mehta B. (2018). Nanostructured TiO_2_ thin films sensitized by CeO_2_ as an inexpensive photoanode for enhanced photoactivity of water oxidation. J. Alloys Compd..

[cit16] Kusmierek E. (2020). A CeO_2_ semiconductor as a photocatalytic and photoelectrocatalytic material for the remediation of pollutants in industrial wastewater: a review. Catalysts.

[cit17] Fauzi A., Jalil A., Hassan N., Aziz F., Azami M., Hussain I., Saravanan R., Vo D.-V. (2022). A critical review on relationship of CeO_2_-based photocatalyst towards mechanistic degradation of organic pollutant. Chemosphere.

[cit18] Ahlawat A., Dhiman T. K., Solanki P. R., Rana P. S. (2022). Enhanced photocatalytic degradation of p-nitrophenol and phenol red through
synergistic effects of a CeO_2_-TiO_2_ nanocomposite. Catal..

[cit19] Hussen A. (2021). PANI/g-C3N4/CeO2 Nanocomposite for Photodegradation of Diazinon in Aqueous Solution. Middle East J. appl. Sci. Technol..

[cit20] Xu L., Wang J. (2012). Magnetic nanoscaled Fe_3_O_4_/CeO_2_ composite as an efficient Fenton-like heterogeneous catalyst for degradation of 4-chlorophenol. Environ. Sci. Technol..

[cit21] Imtiaz A., Farrukh M. A., Latif H. (2024). pH-dependent synthesis of CeO_2_-ZnO nanocomposite for enhanced environmental remediation of endosulfan and dimethoate pesticides. J. Mol. Struct..

[cit22] Hassan S. S. M., Kamel A. H., Hassan A. A., Amr A. E.-G. E., El-Naby H. A., Elsayed E. A. (2020). A SnO_2_/CeO_2_ nano-composite catalyst for alizarin dye removal from aqueous solutions. Nanomaterials.

[cit23] Issarapanacheewin S., Wetchakun K., Phanichphant S., Kangwansupamonkon W., Wetchakun N. (2016). Photodegradation of organic dyes by CeO_2_/Bi_2_WO_6_ nanocomposite and its physicochemical properties investigation. Ceram. Int..

[cit24] Akhtar J., Amin N. S., Aris A. (2011). Combined adsorption and catalytic ozonation for removal of sulfamethoxazole using Fe_2_O_3_/CeO_2_ loaded activated carbon. Chem. Eng. J..

[cit25] Wang X., Huang L., Yuan N., Huang P., Du X., Lu X. (2022). Facile fabrication of a novel SPME fiber based on silicone sealant/hollow ZnO@CeO_2_ composite with super-hydrophobicity for the enhanced capture of pesticides from water. Microchem. J..

[cit26] Murugadoss G., Kumar D. D., Kumar M. R., Venkatesh N., Sakthivel P. (2021). Silver decorated CeO_2_ nanoparticles for rapid photocatalytic degradation of textile rose bengal dye. Sci. Rep..

[cit27] Elhussein E. A. A., Şahin S., Bayazit Ş. S. (2018). Preparation of CeO_2_ nanofibers derived from Ce-BTC metal-organic frameworks and its application on pesticide adsorption. J. Mol. Liq..

[cit28] Bagade A., Nagwade P., Nagawade A., Thopate S., Pandit V., Pund S. (2022). Impact of Mg^2+^ substitution on structural, magnetic and optical properties of Cu-Cd ferrites. Mater. Today: Proc..

[cit29] Hassan S. S. M., El-Shalakany H. H., Fathy M. A., Kamel A. H. (2024). A magnetic macroporous α-Fe_2_O_3_/Mn_2_O_3_ nanocomposite as an efficient adsorbent for simple and rapid removal of Pb (II) from wastewater and electronic waste leachate. Environ. Sci. Pollut. Res..

[cit30] Pandit V. R. U., Jadhav G. K. P., Jawale V. M. S., Dubepatil R., Gurao R., Late D. J. (2024). Synthesis and characterization of micro-/nano-α-Fe_2_O_3_ for photocatalytic dye degradation. RSC Adv..

[cit31] AlZubaidi A., Pandit V., Jawale V., Gupta M., Late D. (2025). Ag-Fe_2_O_3_ nanohybrids for photocatalytic degradation and antibacterial activity. Front. Catal..

[cit32] Grouli A., Bachra Y., Damiri F., Pandit V. U., Berrada M. (2023). Removal of Pollutants from Wastewater Using Fe-Doped Hydroxyapatite Encapsulated with Alginate. Biointerface Res. Appl. Chem..

[cit33] Chalasani R., Vasudevan S. (2013). Cyclodextrin-functionalized Fe_3_O_4_@TiO_2_: reusable, magnetic nanoparticles for photocatalytic degradation of endocrine-disrupting chemicals in water supplies. ACS Nano.

[cit34] Delice S., Isik M., Gasanly N. (2024). Temperature-dependent tuning of band gap of Fe_3_O_4_ nanoparticles for optoelectronic applications. Chem. Phys. Lett..

[cit35] Younis A., Chu D., Li S. (2016). Cerium oxide nanostructures and their applications. Funct. Nanomater..

[cit36] Bhuyan D., Arbuj S. S., Saikia L. (2015). Template-free synthesis of Fe_3_O_4_ nanorod bundles and their highly efficient peroxidase mimetic activity for the degradation of organic dye pollutants with H_2_O_2_. New J. Chem..

[cit37] Pandit V. U., Arbuj S. S., Pandit Y. B., Naik S. D., Rane S. B., Mulik U. P., Gosavi S. W., Kale B. B. (2015). Solar light driven dye degradation using novel organo–inorganic (6,13-pentacenequinone/TiO_2_) nanocomposite. RSC Adv..

[cit38] Fathy M. A., Kamel A. H., Hassan S. S. M. (2022). Novel magnetic nickel ferrite nanoparticles modified with poly (aniline-co-o-toluidine) for the removal of hazardous 2, 4-dichlorophenol pollutant from aqueous solutions. RSC Adv..

[cit39] Hossam F., Elseman A. M., Rasly M., Mahani R., Sayed S., Rashad M. (2023). Observation of structural, optical, electrical and magnetic properties of ternary copper-doped CeO_2_/GO/SrTiO_3_ nanocomposites. J. Mater. Sci.: Mater. Electron..

[cit40] de Morais Teixeira E., Bondancia T. J., Teodoro K. B. R., Corrêa A. C., Marconcini J. M., Mattoso L. H. C. (2011). Sugarcane bagasse whiskers: extraction and characterizations. Ind. Crops Prod..

[cit41] Xie A., Liu W., Wang S., Liu X., Zhang J., Yang Y. (2014). Template-free hydrothermal synthesis and CO oxidation properties of flower-like CeO_2_ nanostructures. Mater. Res. Bull..

[cit42] Zhao W., Chen J., Chang X., Guo S., Srinivasakannan C., Chen G., Peng J. (2014). Effect of microwave irradiation on selective heating behavior and magnetic separation characteristics of Panzhihua ilmenite. Appl. Surf. Sci..

[cit43] Farahmandjou M., Zarinkamar M., Firoozabadi T. (2016). Synthesis of Cerium Oxide (CeO_2_) nanoparticles using simple CO-precipitation method. Rev. Mex. Fis..

[cit44] Calvache-Muñoz J., Prado F. A., Tirado L., Daza-Gomez L. C., Cuervo-Ochoa G., Calambas H. L., Rodríguez-Páez J. E. (2019). Structural and optical properties of CeO_2_ nanoparticles synthesized by modified polymer complex method. J. Inorg. Organomet. Polym. Mater..

[cit45] Thommes M., Kaneko K., Neimark A. V., Olivier J. P., Rodriguez-Reinoso F., Rouquerol J., Sing K. S. (2015). Physisorption of gases, with special reference to the evaluation of surface area and pore size distribution (IUPAC Technical Report). Pure Appl. Chem..

[cit46] Cao X., Meng Z., Song E., Sun X., Hu X., Li W., Liu Z., Gao S., Song B. (2022). Co-adsorption capabilities and mechanisms of bentonite enhanced sludge biochar for de-risking norfloxacin and Cu^2+^ contaminated water. Chemosphere.

[cit47] Xu J., Lin H., Su Y., Tang S. (2025). An Experimental Investigation on the Barrier Performance of Complex-Modified Bentonite. Appl. Sci..

[cit48] Liu J., Cui J., Zhao T., Fan S., Zhang C., Hu Q., Hou X. (2019). Fe_3_O_4_-CeO_2_ loaded on modified activated carbon as efficient heterogeneous catalyst. Colloids Surf., A.

[cit49] López-Benítez A., Guevara-Lara A., Domínguez-Crespo M. A., Andraca-Adame J. A., Torres-Huerta A. M. (2024). Concentrations of organochlorine, organophosphorus, and pyrethroid pesticides in rivers worldwide (2014–2024): A review. Sustainability.

[cit50] El-Mekkawi H., Diab M., Zaki M., Hassan A. (2009). Determination of chlorinated organic pesticide residues in water, sediments, and fish from private fish farms at Abbassa and Sahl Al-Husainia, Shakia Governorate. Aust. J. Basic Appl. Sci..

[cit51] Gong X.-L., Lu H.-Q., Li K., Li W. (2022). Effective adsorption of crystal violet dye on sugarcane bagasse–bentonite/sodium alginate composite aerogel: Characterisation, experiments, and advanced modelling. Sep. Purif. Technol..

[cit52] Hassan S. S. M., Mohamed N. R., Saad M. M., Salem A. M., Ibrahim Y. H., Elshakour A. A., Fathy M. A. (2025). A novel non-woven fabric sandwich filter with activated carbon/polypyrrole nanocomposite for the removal of CO, SO_2_ and NOx emitted from gasoline engines. Fuel.

[cit53] Hassan S. S. M., Mohamed N. R., Saad M. M., Ibrahim Y. H., Elshakour A. A., Fathy M. A. (2025). Eco-Friendly Removal and IoT-Based Monitoring of CO_2_ Emissions Released from Gasoline Engines Using a Novel Compact Nomex/Activated Carbon Sandwich Filter. Polymers.

[cit54] Singh A. K. , Pandit V. U., Sonawane S. L., Bio-based raw materials for preparation of carbon nanostructures, Bio-derived Carbon Nanostructures, Elsevier, 2024, pp. 25–63

[cit55] Šťastný M., Bavol D., Tolasz J., Bezdička P., Čundrle J., Kormunda M., Dimitrov I., Janoš P., Kirakci K., Henych J. (2025). Interfacial behavior of ceria grown on graphene oxide and its use for hydrolytic and photocatalytic decomposition of bisphenols A, S, and F. Environ. Sci.: Nano.

[cit56] Hassan S. S. M., El-Shazly A., Ismael A., Yehia M., Kamel A., Rashad M. (2023). Enhanced photocatalytic degradation of chlorinated pesticides and polychlorinated biphenyls using Mo–TiO_2_/GO/MS nanocomposite. Opt. Mater..

[cit57] Nosaka Y., Nosaka A. (2016). Understanding hydroxyl radical (•OH) generation processes in photocatalysis. ACS Energy Lett..

[cit58] Rizi N. S., Shahzeydi A., Ghiaci M., Zhang L. (2020). Photocatalytic degradation of cationic and anionic organic pollutants in water via Fe-g-C_3_N_4_/CF as a macroscopic photo-Fenton catalyst under visible light irradiation. J. Environ. Chem. Eng..

